# Trend analysis of the role of circular RNA in goat skeletal muscle development

**DOI:** 10.1186/s12864-020-6649-2

**Published:** 2020-03-10

**Authors:** Yinghui Ling, Qi Zheng, Lu Zhu, Lina Xu, Menghua Sui, Yunhai Zhang, Ya Liu, Fugui Fang, Mingxing Chu, Yuehui Ma, Xiaorong Zhang

**Affiliations:** 1 College of Animal Science and Technology, Anhui Agricultural University, Hefei, China; 2 School of Natural and Environmental Sciences, Newcastle University, Newcastle upon Tyne, UK; 3 Local Animal Genetic Resources Conservation and Biobreeding Laboratory of Anhui province, Hefei, China; 4 Institute of Plant Protection and Agro-Products Safety, Anhui Academy of Agricultural Sciences, Hefei, China; 5 Key Laboratory of Farm Animal Genetic Resources and Germplasm Innovation of Ministry of Agriculture, Chinese academy of agricultural sciences, Beijing, China

**Keywords:** circRNA, Skeletal muscle, Development, RNA-seq

## Abstract

**Background:**

Circular RNA (circRNA) is produced during the splicing of mRNA (in addition to linear splicing) and is part of the gene regulatory network. The temporal expression patterns the different developmental stages were inseparable from these molecules’ function.

**Results:**

Skeletal muscles of Anhui white goat (AWG) across seven fetal to postnatal development stages were sequenced and 21 RNA sequencing libraries were constructed. We thereby identified 9090 circRNAs and analyzed their molecular properties, temporal expression patterns, and potential functions at the different stages. CircRNAs showed complexities and diversity of formation as the same host gene produces multiple isoforms of these nucleic acids with different expression profiles. The differential expression of 2881 circRNAs (DECs, *P* < 0.05) was identified and four were randomly selected and validated by qPCR. Moreover, 1118 DECs under strict selected (SDECs, |log2^FC^| > 2 and *P-adj value* < 0.01) showed 4 expression trends (Clusters 0, 19, 16 and 18). Cluster 0 molecules had increasing expression at all stages with effects on muscle through metabolism, regulation of enzyme activity, and biosynthesis. Cluster 16 circRNAs had high expression in the early and late stages and are involved in “Wnt signaling pathway”, “AMPK signaling pathway” and others. Cluster 18 molecules were mainly expressed at F120 and participate in “cytoskeletal protein binding”, “Notch signaling pathway” and so on. Cluster 19 circRNAs were down-regulated at all stages and related to muscle structure and development. Lastly, the SDECs divided the period of skeletal muscle development into three transitional stages: stage 1 (F45 to F90), which related to muscle satellite cell proliferation and muscle fiber structure; stage 2 (F90 to B1), in which the attachment of the cytoplasmic surface to the actin cytoskeleton initiates; and stage 3, which involved the “cGMP-PKG signaling pathway”. Moreover, the paraffin sections messages also validated that there are three transitional stages of skeletal muscle development.

**Conclusion:**

Our current study provides a catalog of goat muscle-related circRNAs that can stratify skeletal muscle development fetus 45 days to newborn 90 days into three developmental stages. These findings better our understanding of functional transitions during mammalian muscle development.

## Introduction

In the past decade, newly identified or rediscovered non-coding RNAs have been associated with striating muscle tissue development and disease [1, 2]. Circular RNA (circRNA) represents a novel class of ncRNAs with a covalent closed-loop structure and no 5′ cap or 3′ poly(A) tail [3]. With the advent of next-generation RNA sequencing (RNA-Seq) and bioinformatics methods, the biological characteristics of circRNAs have gradually been revealed. These molecules are widely expressed but individual circRNAs are specifically active and resistant to RNase R digestion in different cells, tissues and at various developmental stages [4–6]. The biological roles and functions of different circRNAs are also continuously being revealed. They could display as sponge miRNAs [7], regulate gene transcription [8], interact with RNA-binding proteins [9], regulate mRNA stability [8], and even in some cases are translated into polypeptides [10].

Skeletal muscle is derived from embryonic mesoderm precursor cells and the main component of meat produced commercially from mammals [11, 12]. Skeletal muscle has also become an important material for studying mammalian-specific cell differentiation and proliferation mechanisms [13]. In this regard, the *longissimus dorsi* muscle is the largest part of the ridge of the spine and is high economic relevance for fresh and cured meat production [14]. Numerous studies to date have shown that specific circRNAs regulate cell biological processes in skeletal muscle through a variety of gene regulatory mechanisms, including circZNF609, circFGFR4, circLMO7 and others [15–17]. Anhui white goats (AWGs), a native breed of domestic goat (*Capra hircus*), are among the most important commercially farmed animals and have attracted increasing attention as a viable source of meat production. Which is closely related to fetal muscle development. Hence, changes in goat skeletal muscle formation and the underlying molecular mechanisms of this during prenatal and postnatal development are critical considerations for future commercial meat outputs.

In our current study, we evaluated 7 developmental stages of goat skeletal muscle development. We analyzed caesarean-delivered fetuses from AWGs which had been pregnant for 45 (F45), 65 (F65), 90 (F90), 120 (F120) and 135 (F135) days, kids at 1 day (B1) and at 90 days (B90) after birth were used for RNA-seq analysis. We used these goat tissues to systematically identify the molecular characteristics, temporal dynamic expression patterns and potential functions of goat skeletal muscle circRNAs. To the best of our knowledge, this is the first study of muscle circRNA functions in goat. Our findings provided a better understanding of the molecular roles of mammalian circRNAs and of the mechanisms operating at different stages of skeletal muscle development.

## Results

### Overview of circRNA sequencing

**Fig. 1:**
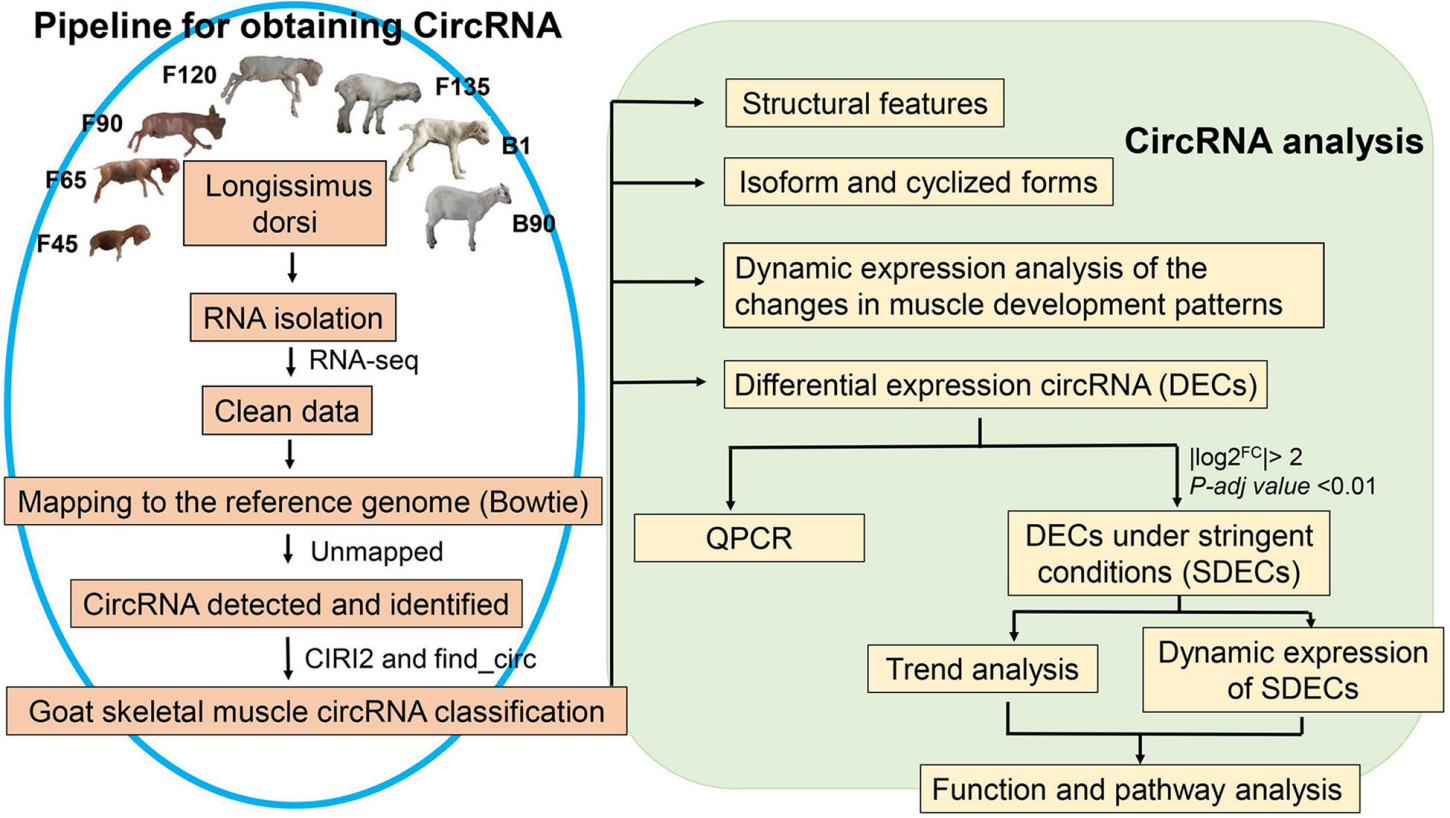
The method pipeline and experimental design used to identify circRNAs involved in goat skeletal muscle. The pictures of animals in Figure 1 were taken by ourselves, and we have the copyright of the original picture

The *longissimus dorsi* from AWGs at 7 different developmental stages were subjected to total RNA-seq to analyze the circRNA profiles of goat skeletal muscle. These stages analyzed included caesarean-delivered fetuses from animals which had been pregnant for 45 (F45), 65 (F65), 90 (F90), 120 (F120), and 135 (F135) days, and from kids born within 24 h (B1) and 90 days old (B90). Each of these stages contained 3 repetitions for a total of 21 skeletal muscle samples. (Fig. 1). The average sample sequencing depth of the 21 cDNA libraries was 109.09 million clean reads, accounting for 96.4% of the raw data (Table S1). Based on the FASTQ file generated, 9090 unique circRNAs were identified by find_circ [18] and CIRI2 [19], which contained CT-AG sliced sites and a read count ≥2 in each sample (Table S2).

### CircRNA isoforms and cyclized form diversity

**Fig. 2:**
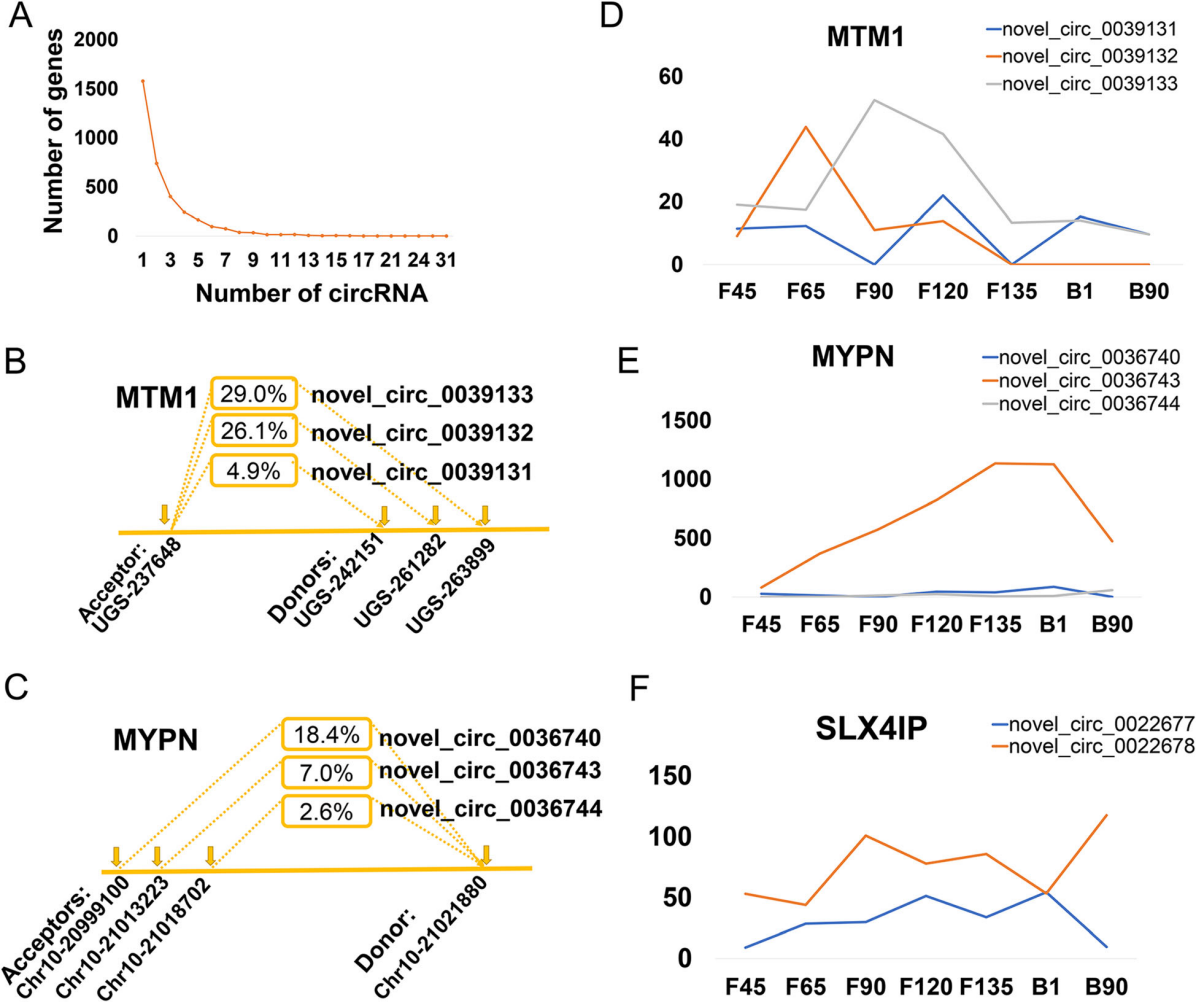
Examples of circRNA isoforms identified in goat. **a** Distribution of circular RNAs among host genes in the goat. **b** Three receptors and one donor position derived from *MTM1* in the genome. **c** Three donors and one receptor position derived from *MYPN* in the genome. **d-f** *MTM1*, *MYPN* and *SLX4IP* expression in the seven stages of goat skeletal muscle development

*Capra hircus* is not included in circBase or PlantcircBase and the identified circRNAs were therefore treated as novel (Table S2). The 8864 identified circRNAs were derived from 3458 loci, and although most genes (67.1%) produced 1 to 2 of these nucleic acids, some produced a variety of distinct cyclides (Fig. 2a). Interestingly, 66.5% of the molecules were formed by host genes that produced multiple circRNAs among those identified. Strikingly, the centrosome forming the backbone *centrosomal protein 112* (*CEP112*) may produce 31 distinct circRNAs. The identification of circRNA isoforms provided a further understanding of the biological mechanisms and modality of circRNA production.

The circRNAs spliced at the same locus contain a splicing signal that was usually composed of the GT dinucleotide at the 5′ end (splice donor) and the AG dinucleotide at the 3′ end (splice acceptor) of the major spliceosome. For a given AG receptor splice site, there was typically more than one GT donor site. For instance, *myotubularin 1* (*MTM1*), which was involved in myotube development, produced three circRNA isoforms (Fig. 2b). The cyclic fraction (the ratio of circular isoforms to all transcripts from the same locus) of the three isoforms (novel_circ_0039131, novel_circ_0039132, novel_circ_0039133) was 4.9, 26.1 and 29.0% with 4503, 23,634 and 26,251 bp in length, respectively. Analogously, many circRNAs had different receptors but share the same donor. For example, the host gene *myopalladin* (*MYPN*) that produced three circRNA isoforms that had three different receptors and shared one donor, Chr10–21,021,880 (Fig. 2c). This analysis indicated the presence of alternate and staggered splicing events in which the same splice site might participate in multiple forward and backward splicing reactions, or at adjacent exons or distal skipped exons.

To reveal the expression profiles of different circRNAs derived from the same host gene, we explored the expression abundance of circRNA isoforms. Different circRNA isoform expression profiles were observed at various developmental stages. Among the three circRNA isoforms of *MTM1* mentioned above, novel_circ_0039132 was found to be highly expressed at F65 but not expressed after F135, whereas novel_circ_0039133 and novel_circ_0039131 reached their highest levels at F90 and F120, respectively (Fig. 2d). For the circRNA isoforms produced by *MYPN*, novel_circ_0036743 was found to gradually increase from F40 to F135, and then begin to decrease, and to reach higher levels at all stages compared to the other two isoforms (Fig. 2e). Interestingly, the two circRNA isoforms of *SLX4 interacting protein gene* (*SLX4IP*) showed a completely opposite expression trend at all stages (Fig. 2f). These results added to our understanding of the diversity and functional complexity of circRNAs.

### Characteristics and expression patterns of the goat skeletal muscle circRNAs

**Fig. 3:**
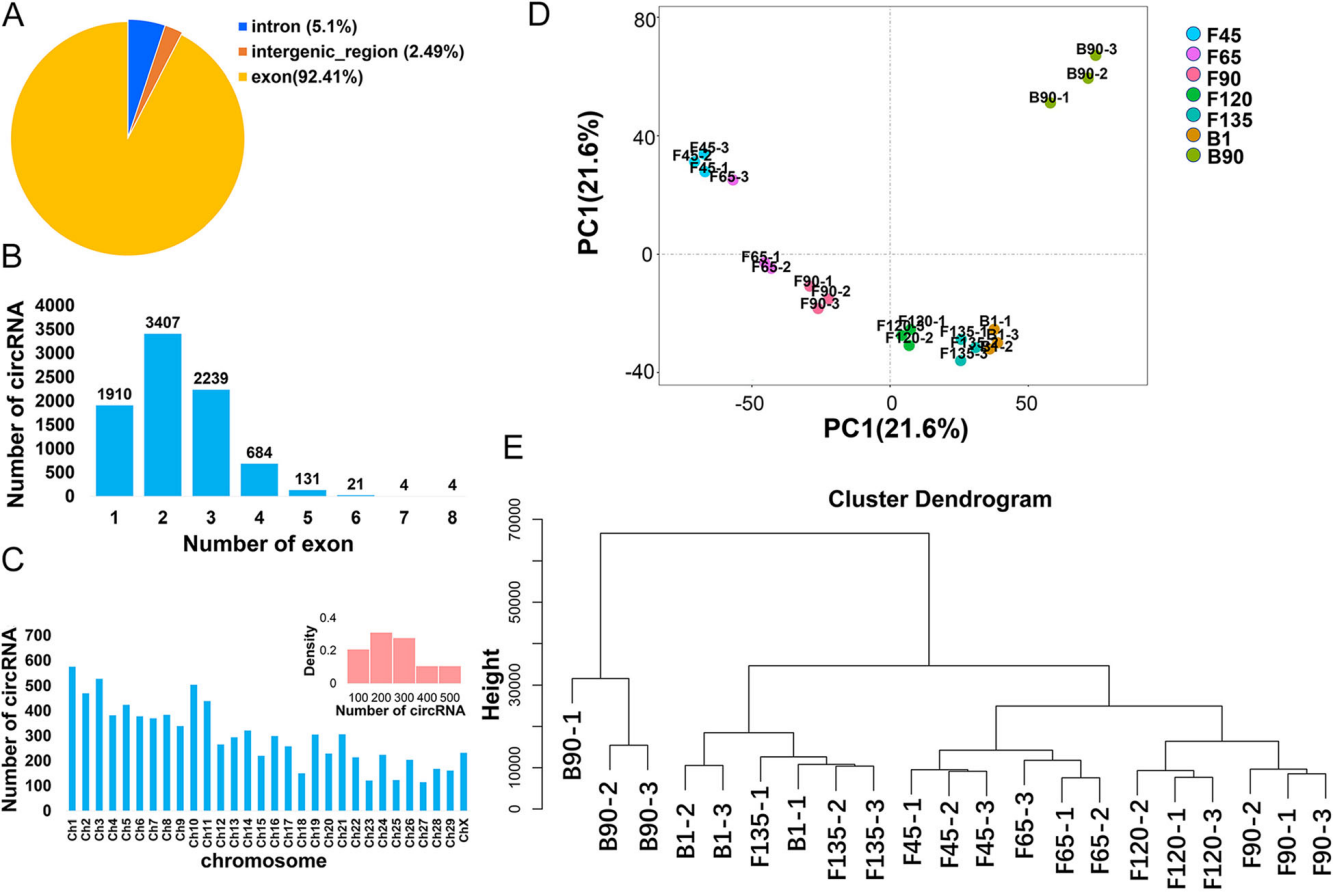
Characterization and expression patterns of circRNAs in goat skeletal muscle. **a** Types of circRNAs in goat skeletal muscle. **b** Number of exons contained in goat circRNAs. **c** Distribution of all identified circRNAs on goat chromosomes. The histogram indicates the circRNA density of each chromosome. **d** Principal component analysis (PCA) of circRNA in 21 goat skeletal muscle samples. Similar colors represent the same phase. Arrows indicate the direction of progression between successive muscle stages. **e** Unsupervised clustering of all circRNAs identified in goat skeletal muscle

To perform a comprehensive analysis of goat skeletal muscle circRNAs, we firstly identified their features. The total panel of 9090 circRNAs were found to be mainly spliced by exons, introns and intergenic regions, with 8400 (92.4%) of these molecules derived from exons (Fig. 3a). All of the goat skeletal muscle circRNAs in 21 libraries consisted of less than 8 exons; 9063 (99.7%) circRNAs spanned 1 to 5 exons (average 2.26), and single exon formation accounted for 22.7% (Fig. 3b). We found that 8783 of the identified circRNAs were unevenly transcribed from the 29 autosome pairs in the goat, 232 were derived from the X chromosome, and the remaining 75 were produced from the unplaced genomic scaffold (Table S2). There were no circRNAs found to be derived from the Y chromosome. Chromosomes 1, 3, and 10 produced more circRNAs (> 500) than any others (Fig. 3c).

We next performed principal component analysis (PCA) on all identified goat skeletal muscle circRNAs to better understand those obtained from the data sets (Fig. 3d). Data from three samples at each stage were pooled, and the order of development could be accurately captured from F45 to B90. We observed that the expression patterns from F45 to B1 were similar, whereas a single cluster appeared after birth, and F45 and F65, and F135 and B1, could be clustered together. Similar expression clustering was found using hierarchical clustering analysis (Fig. 3e). This data may suggest that goat skeletal muscle developmental transitions occur during these stages.

### Dynamic differential expression of circRNAs

**Fig. 4:**
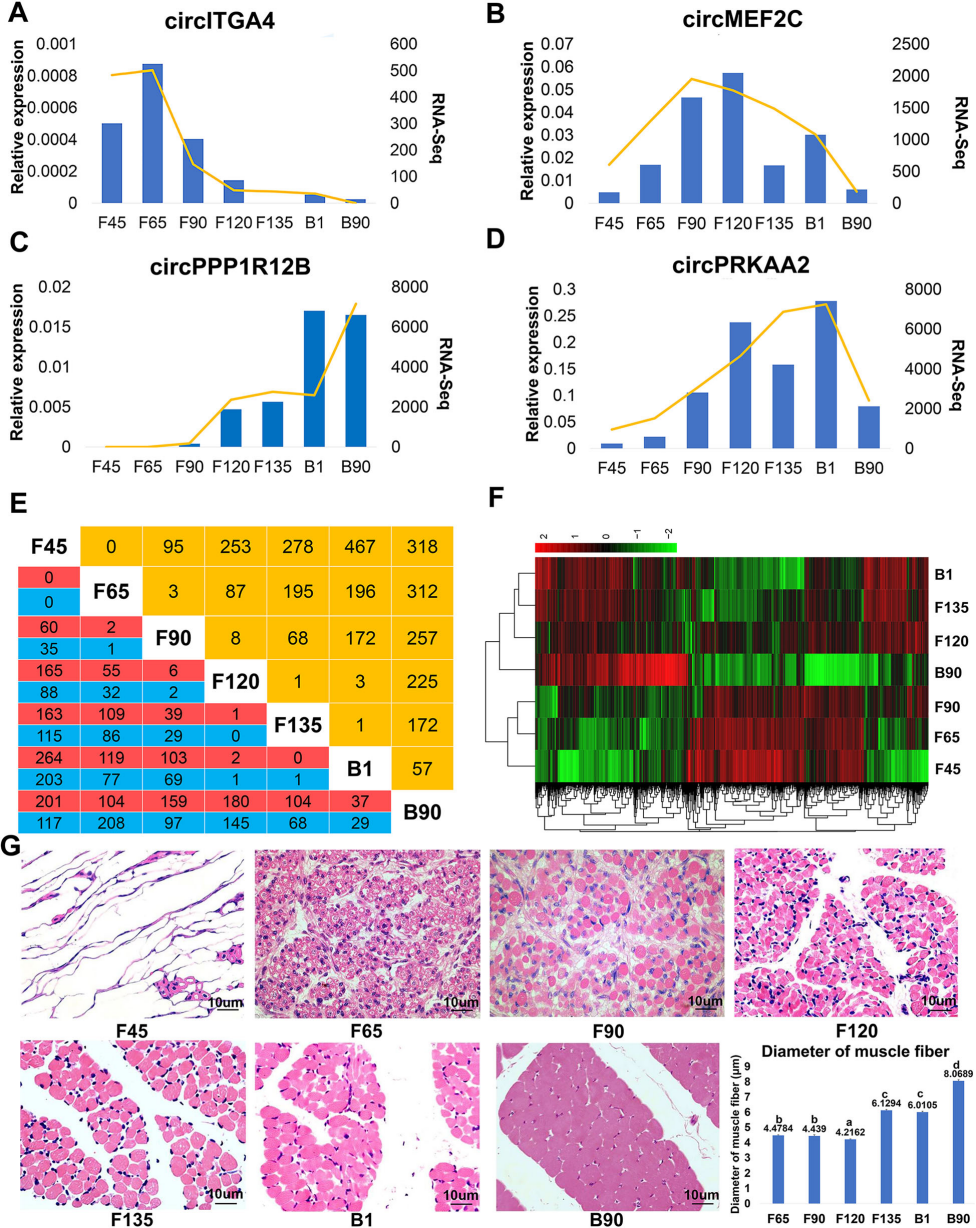
Dynamic differential expression of circRNAs in goat skeletal muscle. **a-d** QPCR (Bar chart, blue) and RNA-seq expression (Line chart, orange) validation of the indicated goat skeletal muscle circRNAs. **e** Number of differentially expressed goat skeletal muscle circRNAs (SDECs) determined under stringent conditions (|log2^FoldChange^ | > 2 and *P- adj value* < 0.01) showing up- (red) or down- (blue) regulation during development. Orange: total number of differentially expressed genes between two stages. **f** SDEC abundance heat maps in the seven development stages of goat skeletal muscle. **g** H&E staining of paraffin sections for the seven development stages of goat skeletal muscle

To further elucidate the regulation of circRNA in skeletal muscle, we performed expression profiling at the seven developmental stages under study. A total of 2881 differentially expressed circRNAs (DECs, *P-adj* < 0.05) were identified during all stages (Table S3). DECs were compared across the 29 goat chromosomes and chromosome 1 was found to be the highest producer (190 DECs) (Fig. S1A). It was thus speculated that chromosome 1 makes a major contribution to the role of circRNAs in the growth of skeletal muscle. The 10 most abundantly expressed circRNAs in goat skeletal muscle were also of great interest (Fig. S1B). The host genes of the most abundantly expressed circRNAs identified by our present analysis, such as novel_circ UBE3A, novel_circMYBPC1, and novel_circLMO7 were primarily associated with muscle growth and muscle myopathy. To verify these results, we randomly selected 4 DECs for qPCR analysis at each of the 7 developmental stages of goat skeletal muscle. The results of these assays were consistent with the expression trends calculated from the RNA-seq data (Fig. 4a-d).

To further explore functional shifts in the circRNAs during goat skeletal muscle development, we assessed 1118 DECs under stringent conditions (SDECs) (|log2^FC^| > 2 and *P-adj* < 0.01) between the different developmental time points (Fig. 4e, Table S4). It was notable that we observed no major goat skeletal muscle SDECs in all but two consecutive stages prior to birth, with the 8 SDECs showing the maximum difference detected between the F90 and F120 stage. The stages from F45 to F65, and F65 to F90 both generated three SDECs, whereas 95 SDECs were generated from F45 to F90. Similarly, there was no significant circRNA expression differences from F120 to B1. The biggest changes were detected between B1 and B90 in which 57 SDECs were evident, 37 of which were up-regulated and 29 were down-regulated. Hence, we defined F45 to F90 as the first functional transition stage (Stage 1) of goat skeletal muscle development, F90 to B1 as the second stage (Stage 2), with the third stage (Stage 3) occurring after birth. Our expression spectrum clustering analysis of SDECs was consistent with this functional transition staging (Fig. 4f). Paraffin section staining also verified three transitional stages of goat skeletal muscle development (Fig. 4g). Primary muscle fibers and secondary muscle fibers were observed in the F65 and F90 stages, in which muscle cells were fused. F120 only observed secondary muscle fibers, and these fibers showed a similar morphology from F120 to B1. Furthermore, muscle fiber diameter data showed that the F120 muscle fiber diameter was significantly smaller (*P* < 0.05) than the other stages. This may be due to the fact that the F120 has completed fusion and the entire muscle cell in the fusion is larger than the muscle cells that complete the fusion. And Similar to the RNA-seq, B90 produced significant differences (*P* < 0.05) with other stages (Fig. 4g).

### Functional and pathway analysis of SDEC in transition stages

**Fig. 5:**
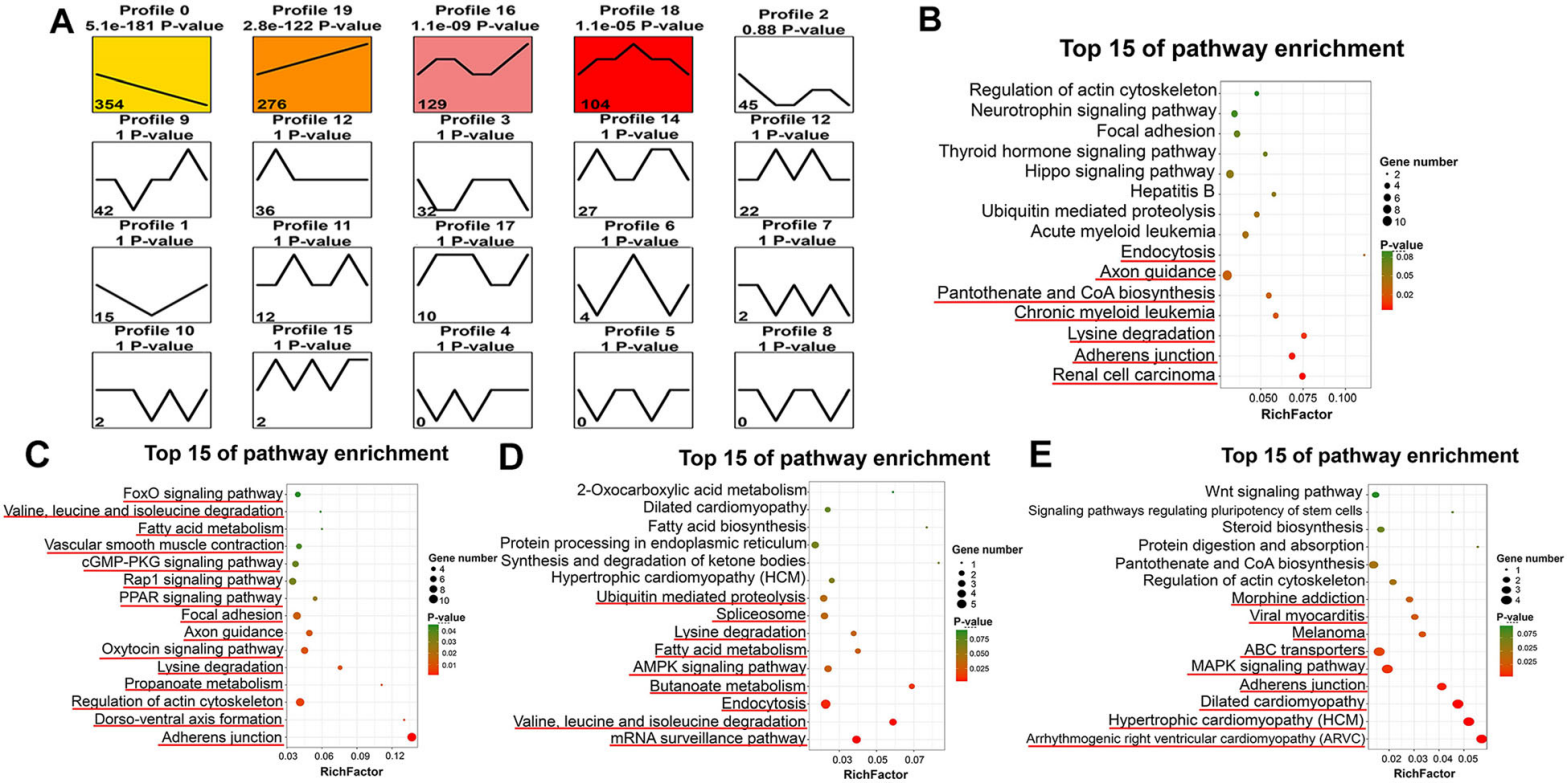
Dynamic differential expression of circRNAs in goat skeletal muscle. **a** Trend analysis of SDECs. Color denotes enrichment. **b-e** Top 15 enriched pathway terms for the goat skeletal muscle circRNAs from modules 1 to 4

We obtained a more comprehensive picture of the dynamic changes in SDEC levels during goat skeletal muscle development through trend analysis. All of the SDECs we analyzed were clustered into 20 expression patterns, with clusters 0, 16, 18, 19 being significantly enriched (Fig. 5a). Clusters 0, 16, 18, and 19 contained 345, 129, 104, and 276 genes, respectively. Using the functions of the circRNA host genes, we predicted the functions of the expression patterns in these four clusters. Cluster 0 (345 SDECs) contained the most SDECs and showed increased expression of these circRNAs from F45 to B90. This cluster was enriched in 162 GO terms, of which 43 are related to the metabolism of substances, 22 to enzyme activity, 22 to biosynthesis and 22 are material-binding related (Table S5), including “transferase activity”, “carbohydrate derivative metabolic process”, “carbohydrate derivative biosynthetic process” and others. Cluster 0 was also enriched in pathways related to muscle function, such as “adherens junction”, “pantothenate and CoA biosynthesis” and “endocytosis” (Fig. 5b). Cluster 19 (276 SDECs) contained circRNAs that were down-regulated at all stages of goat skeletal muscle development was enriched in 94 GO term, among which were those related to muscle development, such as “developmental process”, “BAF-type complex” and others (Fig. 5c, Table S6). Interestingly, cluster 16 (129 SDECs) was found to be enriched in 67 functions, 4 of which were related to the positive regulation of the Wnt signaling pathway that is inseparable from muscle differentiation, development and regeneration (Table S7). Our pathway analysis of cluster 16 also showed it to be enriched in the AMPK signaling pathway associated with muscle hypertrophy (Fig. 5d). In addition, the host genes in cluster 18 (104 SDECs), that had highly expressed circRNAs in stage F120, were involved in “Notch signaling pathway”, “inositol biosynthetic process”, “cytoskeletal protein binding” and others, which were related to muscle structure, proliferation and regeneration (Table S8). Pathway analysis of cluster 18 also revealed that the host genes were significantly associated with “MAPK signaling pathway” and “adherens junction”. These data indicated that RNA cyclization is indispensable for the regulation of mammalian muscle development (Fig. 5e).

We next focused on the SDECs between F45 and F90 (Stage 1, 97 SDECs), F90 to B1 (Stage 2, 196 SDECs) and B1 to B90 (Stage 3, 57 SDECs). Remarkably, there were SDECs common to all three transitional stages, with 79% (77 of 97 total), 88.3% (173 of 196 total) and 77.2% (44 of 57 total) of these circRNAs found to be exclusively expressed at each stage, respectively (Fig. S2). None of the uniquely expressed SDECs in these three stages showed any significant functional enrichment, but they participated in different signaling pathways. Interestingly, the host genes *FGFR1*, *LPAR1*, and *PDGFC* of Stage 1 were found to be involved in “Rap1 signaling pathways”, “PI3K-Akt signaling pathway”, “regulation of actin cytoskeleton” and other signaling pathways that correlated with muscle satellite cell proliferation and muscle fiber structure (Table S9). The host genes from Stage 2 were enriched in eight signaling pathways with an involvement in acid metabolism and the attachment of the cytoplasmic surface to the actin cytoskeleton (Table S10). In Stage 3, which was post-birth, included host genes *VDAC1* and *MEF2C* were involved in the “cGMP-PKG signaling pathway”, and *PANK3* and *PDE8A* were involved in “pantothenate and CoA biosynthesis” (Table S11). These circRNAs may represent the initiation/termination of growth and/or physiological processes at a particular developmental stage in skeletal muscle.

## Discussion

With the development of RNA-seq for genome-wide analysis, circRNA have been found to be more abundant in the animal transcriptome than previously thought, and thousands of these molecules have now been identified in human [20], mouse [21], pig [22] and other species. Gruner et al., previously conducted sequencing analysis with HiSeq 2500 and identified 6791 circRNAs in the heart, cerebral cortex and hippocampus of the mouse at different ages [21]. Furthermore, based on 24 skeletal muscle samples from differently aged animals, Kotb’s team identified 12,007 Rhesus monkey (*Macaca mulatta*) circRNAs [23]. Recently, 21,813 circRNAs were identified during the pre-receptive to receptive phases in goat endometrium development [24]. These prior findings have indicated that circRNAs were abundant and yet to be fully explored, and that they played an important role in mammalian development. In our current study, we have identified 9090 circRNAs in goal skeletal muscle over five fetal developmental stages (45-, 65-, 90-, 120- and 135- days fetus) and in 1- and 90-day-old kids. Because circRNAs were expressed in a highly spatiotemporal manner, studies of these molecules across different developmental stages and in different tissues had been critical [25].

CircRNAs, as regulators of gene expression, have similar structural features. Most of these nucleic acids consisted of fewer than 5 exons in goat skeletal muscle, which was consistent with the exon numbers in pig circRNAs [26]. However, circRNAs with a single exon were more prominent in goat skeletal muscle compared with that in pigs and mice, which may be a characteristic of skeletal muscle circRNA formation in goats [26, 27]. The most abundant circRNAs reported previously were derived from chromosome 6 in 9 tissues and 3 stages of skeletal muscle development in pigs [26]. We found in our current analysis that goat circRNAs were mainly produced from chromosome 1, regardless of whether they were differentially expressed. This indicated that chromosome 1 made an indispensable contribution to the role of circRNAs in the development of goat skeletal muscle.

Secondary (fetal) myogenesis relies on the fusion of fetal progenitor cells which produce secondary muscle fibers with no net increase in the number of muscle fibers after birth [28, 29]. Additionally, the skeletal muscle mass increased during postnatal animal development via hypertrophy [30]. The function of circRNAs in goat skeletal muscle development before and after birth remains unclear. All of the estimated 1118 SDECs in our current analyses could be mainly classified into four modules (cluster 0, 16, 18, 19) in accordance with their expression profiles. The functions associated with these clusters may be associated with the prenatal and postnatal growth of goat skeletal muscle. The GO and KEGG enrichment for the circRNAs in cluster 0, the expression of which gradually raised with the developmental progression in goat skeletal muscle, mainly involved substance synthesis, metabolism and enzyme activity. For example, novel_circ_0025911 was generated from the host gene *AKT3* which functions in transferase activity and suppressed C2C12 differentiation when overexpressed [31]. The functions and pathways associated with the cluster 19 circRNAs, that were found to be down-regulated at all stages of development in goat skeletal muscle, appeared to be associated mainly with muscle proliferation and differentiation. The host gene for novel_circ_0034197 and novel_circ_0031011 in this cluster was *MEF2C*, which maintains sarcomere integrity and skeletal muscle postnatal maturation [32, 33]. The *ZNRF1* gene in cluster 16, which is related to the regulation of the Wnt signaling pathway, produces novel_circ_0028110. Canonical Wnt signaling induces satellite-cell proliferation during adult skeletal muscle regeneration [34, 35]. The novel_circ_0027226 assigned to cluster 18 is produced by the host gene *FGF2,* which functions in the MAPK signaling pathway. *FGF2* is upstream of MAPK signaling, which mainly regulates myocyte hypertrophy and development [36–38]. Taken together, these results demonstrate that specific circRNAs perform their functions at different stages of skeletal muscle development.

Based on the observed circRNA profiles, we designated three periods of functional transition in goat skeletal muscle development. These three phases were further validated by the expression data from paraffin sections. Our present study is the first to systematically identify different circRNAs that function specifically at different developmental stages of goat skeletal muscle. These data provide new insights into the role of circRNAs in the evolution of muscle development at different stages. The unique circRNAs that operate at the three functional transition periods in goat skeletal muscle may represent the beginning/termination of a certain physiological and/or growth process at a particular stage. For example, circPDGFC, circTTN, circITGA4, and circLPAR1 that are expressed in stage 1 (from F45 to F90) take part in the “PI3K-Akt signaling pathway”, “regulation of actin cytoskeleton” and other processes that relate to muscle developmental, formation and structure [39–41]. Stage 2 circRNAs, operating from F90 to B1, are involved in acid metabolism and the attachment of the cytoplasmic surface to the actin cytoskeleton, including circACTN2, circMAPK1 and others. Furthermore, circMEF2C, circVDAC1 from stage 3 are involved in the cGMP-PKG signaling pathway which plays a role in muscle contraction regulation [42].

## Conclusion

We here describe for the first time a 7-stage circRNA expression profile in goat skeletal muscle development by RNA-seq and characterize the temporal patterns of SDECs. The seven stage circRNA in goat identified four major expression patterns and three skeletal muscle function transition stages (F45 to F90, F90 to B1, and B1 to B90) based on clustering and differential expression analysis. We will validate these findings in future studies to gain a deeper understanding of the functional roles of the circRNAs in mammalian muscle development.

## Materials and methods

### Sample collection

All experimental Anhui white goats (AWGs) were managed and raised on a farm managed by the Hefei Boda Animal Husbandry Technology Development Co., Ltd. (Anhui, China). EAZI-Breed CIDR (CIDR, Hamilton, New Zealand) was used to treat female goats for estrus. CIDR was removed after embedding 12 days, and 1 ml of prostaglandin was injected into the neck muscle of each female goat. Fetuses were obtained from goats that had been pregnant for 45, 65, 90, 120 and 135 days (F45, F65, F90, F120, F135) by cesarean section. Before sample collection, the all experimental samples (include female goats, fetuses and kids) were injected with Jingsongling (2,4-xylyl xylazole, lot number 030725, produced by Shandong Zibo Veterinary Medicine Factory, Shandong, China) in the hips at a dose of 2 mg / kg for deep anesthesia. Under complete anesthesia, all experimental samples were killed by arterial bleeding with the help of slaughterhouse professionals. (this method complies with euthanasia guide of the Chinese Society of Laboratory Animals, No. T / CALAS 31–2017). Kids born naturally at 1 day and 90 days (B1 and B90) of age were also collected, then their *longissimus dorsi* muscles were harvested as experimental samples. A total of 21 goats were used as experimental animals in the seven stages, and each stage had three biological replicates. Two samples were collected from each goat for a total of 42 skeletal muscle samples. All samples were divided into two groups, each group containing three biological replicates of seven stage. One group of samples was frozen in liquid nitrogen until RNA extraction was needed, and the other was placed in an Environmentally Friendly GD Fixative (Wuhan, Hubei, China) to prepare paraffin sections.

### Paraffin section and staining

The skeletal muscle mass (1 cm × 1 cm × 1.5 cm) in the muscle-specific fixative was paraffin-embedded by a conventional method. The embedded paraffin was continuously sliced at a thickness of 5 μm. H&E staining was performed by conventional staining method, in which time of hematoxylin staining was about 4 min, and 0.5% eosin staining was stained for 3 min. Three slices were selected for each sample and taken with the Olympus BX51 (Tokyo, Japan) image acquisition system. Skeletal muscle cell morphology was observed with a 40 × 10 microscope. The skeletal muscle fiber diameter was measured using Image Pro® 6.0 (Image Pro®, Media Cybernetics Inc.). All diameter data were processed using Windows version of SPSS 21.0, and One-way ANOVA was used for calculations. The analysis results include means ± SEM. When the *P-*value is < 0.05, the data of this group was statistically significant.

### RNA extraction and detection

RNA was extracted from 21 skeletal muscle samples using TRIzol reagent (Invitrogen, Carlsbad, CA) according to the manufacturer’s instructions. Next, the 1% agarose gel was used to detect whether the obtained RNA was degraded or contaminated. And NanoPhotometer® spectrophotometer (IMPLEN, CA, USA) measured the purity of the remaining RNA extract. Subsequent RNA extract detection concentrations were performed using Qubit® RNA Assay Kit and Qubit® 2.0 Fluorometer (Life Technologies, CA, USA) and RNA intact under the Agilent 2100 Bioanalyzer (Agilent Technologies, CA, USA) using a matching kit Sexual assessment. Among them, the test result required that the ratio of RNA with RIN ≥ 7, 28S: 18S ≥ 1.5: 1.

### RNA library preparation and sequencing

Select a qualified total RNA as the starting sample for RNA-seq. The starting amount of RNA is greater than 5μg. The available samples were further processed according to the library construction strategy. The Ribo-Zero™ Magnetic Kit (Epicentre, Madison, Wisconsin) was used to separate and remove rRNA from total RNA, and then digested with Ribonuclease R (Epicentre). Next, the RNA Library Preparation Kit NEBNext® Ultra™ generates sequencing libraries from rRNA-deleted RNA. The short RNA was used as a template, and the first strand of cDNA was synthesized using six base random primers. Then add buffer, dNTPs (replace dTTP with dUTP), RNase H and DNA polymerase I to synthesize the second strand of cDNA. Sequence-specific circRNA libraries were constructed by adding sequencing adapter sequences and EB buffer to elute for end repair, followed by cluster production reactions to obtain sequences for sequencing on the machine. Finally, each library was then sequenced through the Illumina Hiseq 4000 platform.

### Data processing and circRNA identification

The original image data file obtained by sequencing by the sequencing platform was converted into a sequence file, that is, raw data, through base recognition analysis. Then the low-quality sequences in the original sequence data and the adapter sequences needed for sequencing were removed in in-house Perl scripts to obtain clean data, and the GC content was calculated. Reference genome and gene (Assembly accession No. GCF_001704415.1) model annotation files of goat (*Capra hircus*) were downloaded directly from the web of NCBI. Bowtie2 v2.2.8 [43] was used to compare clean data with reference genomes and genes, and then circRNA was screened and identified by find_circ [18] and CIRI2 [19].

### Identification of DECs

First, the number of reads that the identified circRNA was aligned to the reference genome was analyzed. After obtaining the number of reads, use the Bowtie to normalize the sample reads separately [43]. The standardized unit used here is trans per million (TPM). The relative average TPM of each circRNA in the sample was obtained by processing, and the difference analysis was performed for each group using the DESeq R software package (1.10.1). DECs were screened under the condition of *P-*value < 0.05.

### Quantitative RT-PCR validation

GoTaq qPCR Master Mix (A6002, Promega, Madison, WI) and a Real-time Thermal Cycler 5100 (Thermo, USA) were used for qRT-PCR detection to assess the amount of four randomly selected DECs expression in different stages. The same total RNA samples used in the sequencing analysis were used as the template in these amplification reactions. All of the primer pairs (Table S12) were designed using Primer-BLAST in NCBI and synthesized by the Shanghai General Biotech Co. Ltd. The skeletal muscle *ACTB* (*β-actin*) sequence was used as a reference gene to uniformize the expression level of the DECs [44]. The relative expression of circRNA in this data was calculated using 2^−ΔΔCt^. All qRT-PCR data were processed by SPSS 21.0, and One-way ANOVA was used for calculations. The analysis results include means ± SEM. When the *P-*value < 0.05 between the two groups, the two groups were considered to be significantly different.

### Trend analysis

Expression of the identified strict condition DECs were clustered, compared and visualized using the default mode of Stem [45]. By assessing the degree of association between gene expression trends and changes in sample traits, gene groups unrelated to sample changes were isolated. Additionally, the Stem software extracts and color-codes the trends in mainstream gene expression, and thereby ranks important gene expression trends in order of importance from small to large. Genes with a *P-*value < 0.05 identified by Stem were considered to be enriched.

### Function and pathway term enrichment

In the GO function enrichment analysis, all genes are first mapped to each entry in the GO database. Second, according to the number of genes in each term, a hypergeometric test was applied to find out which genes were significantly enriched GO term compared to the entire genome background. For the target gene set, GO analysis was performed using GOseq R package [46]. Furthermore, KOBAS software was used to test the statistical enrichment of host genes of circRNA [47]. We take *P*-value < 0.05 as the threshold, and GO and KEGG terms that met this condition were defined as GO and KEGG terms that were significantly enriched in DEGs.

## Supplementary information

**Additional file 1: Fig supplement 1.** (A) Distribution of SDECs on chromosomes and the histogram in the upper right corner represents the SDECs density of the chromosome. (B) Top differentially expressed circRNAs expressed in seven stages.

**Additional file 2: Fig supplement 2.** Venn chart of SDECs detected in three transitional stages.

**Additional file 3: Table S1.** The summary information of transcriptome.

**Additional file 4: Table S2.** Identification of all circRNAs and their annotations.

**Additional file 5: Table S3.** Differently expressed circRNAs and their annotations during the seven stages.

**Additional file 6: Table S4**. Stringent conditions differential expression of circRNAs and their annotations identified during the seven stages.

**Additional file 7: Table S5.** Functional analysis of cluster 0.

**Additional file 8: Table S6.** Functional analysis of cluster 19.

**Additional file 9: Table S7**. Functional analysis of cluster 16.

**Additional file 10: Table S8**. Functional analysis of cluster 18.

**Additional file 11: Table S9**. KEGG enrichment of stage 1.

**Additional file 12: Table S10**. KEGG enrichment of stage 2.

**Additional file 13: Table S11**. KEGG enrichment of stage 3.

**Additional file 14: Table S12**. Primer pairs used for qRT-PCR amplification.

## Data Availability

The goat (*Capra hircus*) reference genome and gene model is downloaded directly from NCBI Genomic. The corresponding accession number of Sequence Read Archive (SRA) database for this genome is PRJNA340281, and the name is “*Capra hircus* breed: San Clemente Raw sequence reads”. The raw data files obtained from RNA-seq have been uploaded and made public to the NCBI SRA database. The accession number is PRJNA553597, and the name is “sequencing of skeletal muscle transcriptome before and after goat birth”.

## Abbreviations

AWG: Anhui white goat
circRNA: Circular RNA
DEC: Differential expression circRNA
SDEC: DEC under strict selected
F45: Fetus 45 days
F65: Fetus 65 days
F90: Fetus 90 days
F120: Fetus 120 days
F135: Fetus 135 days
B1: Born 1 day
B90: Born 90 days

## References

[1] Ballarino M, Morlando M, Fatica A, Bozzoni I (2016). Non-coding RNAs in muscle differentiation and musculoskeletal disease. J Clin Invest.

[2] Mok GF, Lozano-Velasco E, Munsterberg A (2017). microRNAs in skeletal muscle development. Semin Cell Dev Biol.

[3] Meng S, Zhou H, Feng Z, Xu Z, Tang Y, Li P, Wu M (2017). CircRNA: functions and properties of a novel potential biomarker for cancer. Mol Cancer.

[4] Chen LL (2016). The biogenesis and emerging roles of circular RNAs. Nat Rev Mol Cell Biol.

[5] Boeckel JN, Jae N, Heumuller AW, Chen W, Boon RA, Stellos K, Zeiher AM, John D, Uchida S, Dimmeler S (2015). Identification and characterization of hypoxia-regulated endothelial circular RNA. Circ Res.

[6] Xu K, Chen D, Wang Z, Ma J, Zhou J, Chen N, Lv L, Zheng Y, Hu X, Zhang Y (2018). Annotation and functional clustering of circRNA expression in rhesus macaque brain during aging. Cell discovery.

[7] Hansen TB, Jensen TI, Clausen BH, Bramsen JB, Finsen B, Damgaard CK, Kjems J (2013). Natural RNA circles function as efficient microRNA sponges. Nature.

[8] Li Y, Zheng Q, Bao C, Li S, Guo W, Zhao J, Chen D, Gu J, He X, Huang S (2015). Circular RNA is enriched and stable in exosomes: a promising biomarker for cancer diagnosis. Cell Res.

[9] Qu S, Yang X, Li X, Wang J, Gao Y, Shang R, Sun W, Dou K, Li H (2015). Circular RNA: A new star of noncoding RNAs. Cancer Lett.

[10] Li X, Yang L, Chen LL (2018). The biogenesis, functions, and challenges of circular RNAs. Mol Cell.

[11] Chal J, Pourquie O (2017). Making muscle: skeletal myogenesis in vivo and in vitro. Development (Cambridge, England).

[12] Nandkishore Nitya, Vyas Bhakti, Javali Alok, Ghosh Subho, Sambasivan Ramkumar (2018). Divergent early mesoderm specification underlies distinct head and trunk muscle programmes in vertebrates. Development.

[13] Jana S, Levengood SK, Zhang M (2016). Anisotropic Materials for Skeletal-Muscle-Tissue Engineering. Adv Mater.

[14] Ovilo C, Benitez R, Fernandez A, Nunez Y, Ayuso M, Fernandez AI, Rodriguez C, Isabel B, Rey AI, Lopez-Bote C (2014). Longissimus dorsi transcriptome analysis of purebred and crossbred Iberian pigs differing in muscle characteristics. BMC Genomics.

[15] Legnini I, Di Timoteo G, Rossi F, Morlando M, Briganti F, Sthandier O, Fatica A, Santini T, Andronache A, Wade M (2017). Circ-ZNF609 Is a Circular RNA that Can Be Translated and Functions in Myogenesis. Mol Cell.

[16] Wei X, Li H, Yang J, Hao D, Dong D, Huang Y, Lan X, Plath M, Lei C, Lin F (2017). Circular RNA profiling reveals an abundant circLMO7 that regulates myoblasts differentiation and survival by sponging miR-378a-3p. Cell Death Dis.

[17] Li H, Wei X, Yang J, Dong D, Hao D, Huang Y, Lan X, Plath M, Lei C, Ma Y (2018). circFGFR4 promotes differentiation of myoblasts via binding miR-107 to relieve its inhibition of Wnt3a. Mol Ther Nucleic Acids.

[18] Memczak S, Jens M, Elefsinioti A, Torti F, Krueger J, Rybak A, Maier L, Mackowiak SD, Gregersen LH, Munschauer M (2013). Circular RNAs are a large class of animal RNAs with regulatory potency. Nature.

[19] Gao Y, Zhang J, Zhao F (2018). Circular RNA identification based on multiple seed matching. Brief Bioinform.

[20] Tan WL, Lim BT, Anene-Nzelu CG, Ackers-Johnson M, Dashi A, See K, Tiang Z, Lee DP, Chua WW, Luu TD (2017). A landscape of circular RNA expression in the human heart. Cardiovasc Res.

[21] Gruner H, Cortes-Lopez M, Cooper DA, Bauer M, Miura P (2016). CircRNA accumulation in the aging mouse brain. Sci Rep.

[22] Li A, Huang W, Zhang X, Xie L, Miao X (2018). Identification and characterization of CircRNAs of two pig breeds as a new biomarker in metabolism-related diseases. Cellular Physiol Biochem.

[23] Abdelmohsen K, Panda AC, De S, Grammatikakis I, Kim J, Ding J, Noh JH, Kim KM, Mattison JA, de Cabo R (2015). Circular RNAs in monkey muscle: age-dependent changes. Aging.

[24] Song Y, Zhang L, Liu X, Niu M, Cui J, Che S, Liu Y, An X, Cao B (2019). Analyses of circRNA profiling during the development from pre-receptive to receptive phases in the goat endometrium. J Animal Sci biotechnol.

[25] Veno MT, Hansen TB, Veno ST, Clausen BH, Grebing M, Finsen B, Holm IE, Kjems J (2015). Spatio-temporal regulation of circular RNA expression during porcine embryonic brain development. Genome Biol.

[26] Liang G, Yang Y, Niu G, Tang Z, Li K (2017). Genome-wide profiling of Sus scrofa circular RNAs across nine organs and three developmental stages. DNA Res.

[27] Fan X, Zhang X, Wu X, Guo H, Hu Y, Tang F, Huang Y (2015). Single-cell RNA-seq transcriptome analysis of linear and circular RNAs in mouse preimplantation embryos. Genome Biol.

[28] Wang H, Noulet F, Edom-Vovard F, Tozer S, Le Grand F, Duprez D (2010). Bmp signaling at the tips of skeletal muscles regulates the number of fetal muscle progenitors and satellite cells during development. Dev Cell.

[29] Zhu MJ, Ford SP, Nathanielsz PW, Du M (2004). Effect of maternal nutrient restriction in sheep on the development of fetal skeletal muscle. Biol Reprod.

[30] Schiaffino S, Dyar KA, Ciciliot S, Blaauw B, Sandri M (2013). Mechanisms regulating skeletal muscle growth and atrophy. FEBS J.

[31] Wei W, He HB, Zhang WY, Zhang HX, Bai JB, Liu HZ, Cao JH, Chang KC, Li XY, Zhao SH (2013). miR-29 targets Akt3 to reduce proliferation and facilitate differentiation of myoblasts in skeletal muscle development. Cell Death Dis.

[32] Raices M, Bukata L, Sakuma S, Borlido J, Hernandez LS, Hart DO, D'Angelo MA (2017). Nuclear Pores Regulate Muscle Development and Maintenance by Assembling a Localized Mef2C Complex. Developmental cell.

[33] Potthoff MJ, Arnold MA, McAnally J, Richardson JA, Bassel-Duby R, Olson EN (2007). Regulation of skeletal muscle sarcomere integrity and postnatal muscle function by Mef2c. Mol Cell Biol.

[34] Otto A, Schmidt C, Luke G, Allen S, Valasek P, Muntoni F, Lawrence-Watt D, Patel K (2008). Canonical Wnt signalling induces satellite-cell proliferation during adult skeletal muscle regeneration. J Cell Sci.

[35] Zhang K, Zhang Y, Gu L, Lan M, Liu C, Wang M, Su Y, Ge M, Wang T, Yu Y (2018). Islr regulates canonical Wnt signaling-mediated skeletal muscle regeneration by stabilizing Dishevelled-2 and preventing autophagy. Nat Commun.

[36] House SL, Branch K, Newman G, Doetschman T, Schultz JJ (2005). Cardioprotection induced by cardiac-specific overexpression of fibroblast growth factor-2 is mediated by the MAPK cascade. Am J Phys Heart Circ Phys.

[37] Lawan A, Min K, Zhang L, Canfran-Duque A, Jurczak MJ, Camporez JPG, Nie Y, Gavin TP, Shulman GI, Fernandez-Hernando C (2018). Skeletal muscle-specific deletion of MKP-1 reveals a p38 MAPK/JNK/Akt signaling node that regulates obesity-induced insulin resistance. Diabetes.

[38] Riuzzi F, Sorci G, Sagheddu R, Donato R (2012). HMGB1-RAGE regulates muscle satellite cell homeostasis through p38-MAPK- and myogenin-dependent repression of Pax7 transcription. J Cell Sci.

[39] Svitkina Tatyana (2018). The Actin Cytoskeleton and Actin-Based Motility. Cold Spring Harbor Perspectives in Biology.

[40] Ma M, Wang X, Chen X, Cai R, Chen F, Dong W, Yang G, Pang W (2017). MicroRNA-432 targeting E2F3 and P55PIK inhibits myogenesis through PI3K/AKT/mTOR signaling pathway. RNA Biol.

[41] Zhou BH, Tan PP, Jia LS, Zhao WP, Wang JC, Wang HW (2018). PI3K/AKT signaling pathway involvement in fluoride-induced apoptosis in C2C12cells. Chemosphere.

[42] Morelli A, Filippi S, Sandner P, Fibbi B, Chavalmane AK, Silvestrini E, Sarchielli E, Vignozzi L, Gacci M, Carini M (2009). Vardenafil modulates bladder contractility through cGMP-mediated inhibition of RhoA/rho kinase signaling pathway in spontaneously hypertensive rats. J Sex Med.

[43] Langmead B, Trapnell C, Pop M, Salzberg SL (2009). Ultrafast and memory-efficient alignment of short DNA sequences to the human genome. Genome Biol.

[44] Zhan S, Dong Y, Zhao W, Guo J, Zhong T, Wang L, Li L, Zhang H (2016). Genome-wide identification and characterization of long non-coding RNAs in developmental skeletal muscle of fetal goat. BMC Genomics.

[45] Ernst J, Bar-Joseph Z (2006). STEM: a tool for the analysis of short time series gene expression data. BMC Bioinformatics.

[46] Young MD, Wakefield MJ, Smyth GK, Oshlack A (2010). Gene ontology analysis for RNA-seq: accounting for selection bias. Genome Biol.

[47] Mao X, Cai T, Olyarchuk JG, Wei L (2005). Automated genome annotation and pathway identification using the KEGG Orthology (KO) as a controlled vocabulary. Bioinformatics (Oxford, England).

