# Supplementary figures and images for: Trend analysis of the role of circular RNA in goat skeletal muscle development
Source: BMC Genomics. 2020 Mar 10;21:220. doi: 10.1186/s12864-020-6649-2 (PMC7063781; doi:10.1186/s12864-020-6649-2)

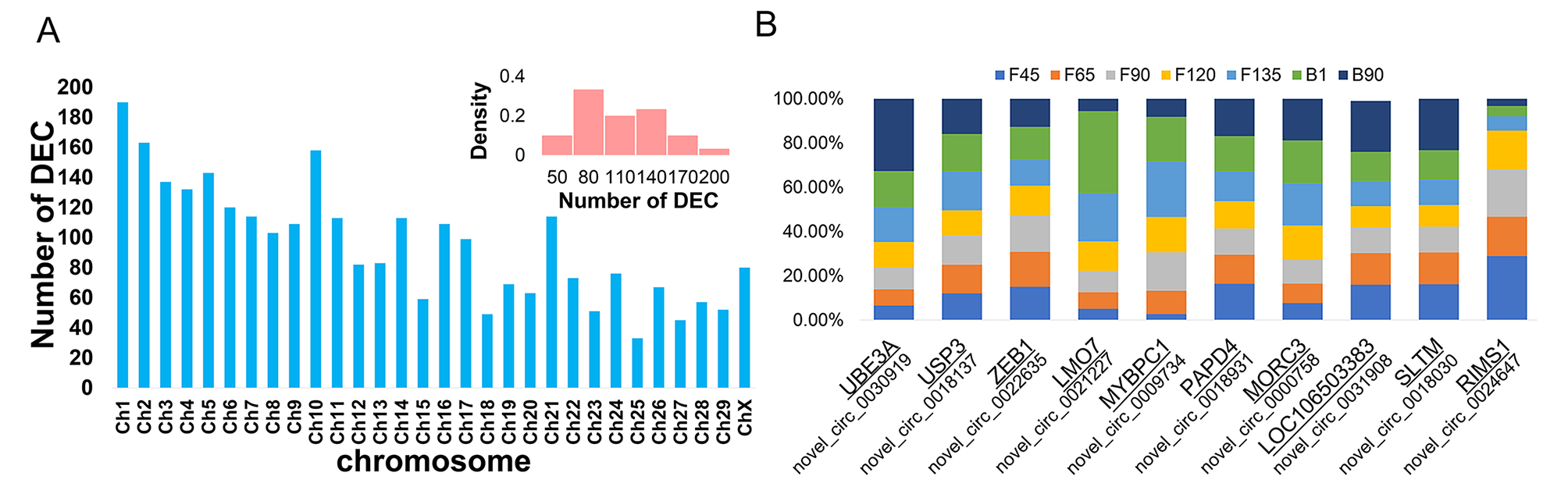

Supplement: Supplementary file 1 — Additional file 1: Fig supplement 1. (A) Distribution of SDECs on chromosomes and the histogram in the upper right corner represents the SDECs density of the chromosome. (B) Top differentially expressed circRNAs expressed in seven stages. [file 12864_2020_6649_MOESM1_ESM.png]

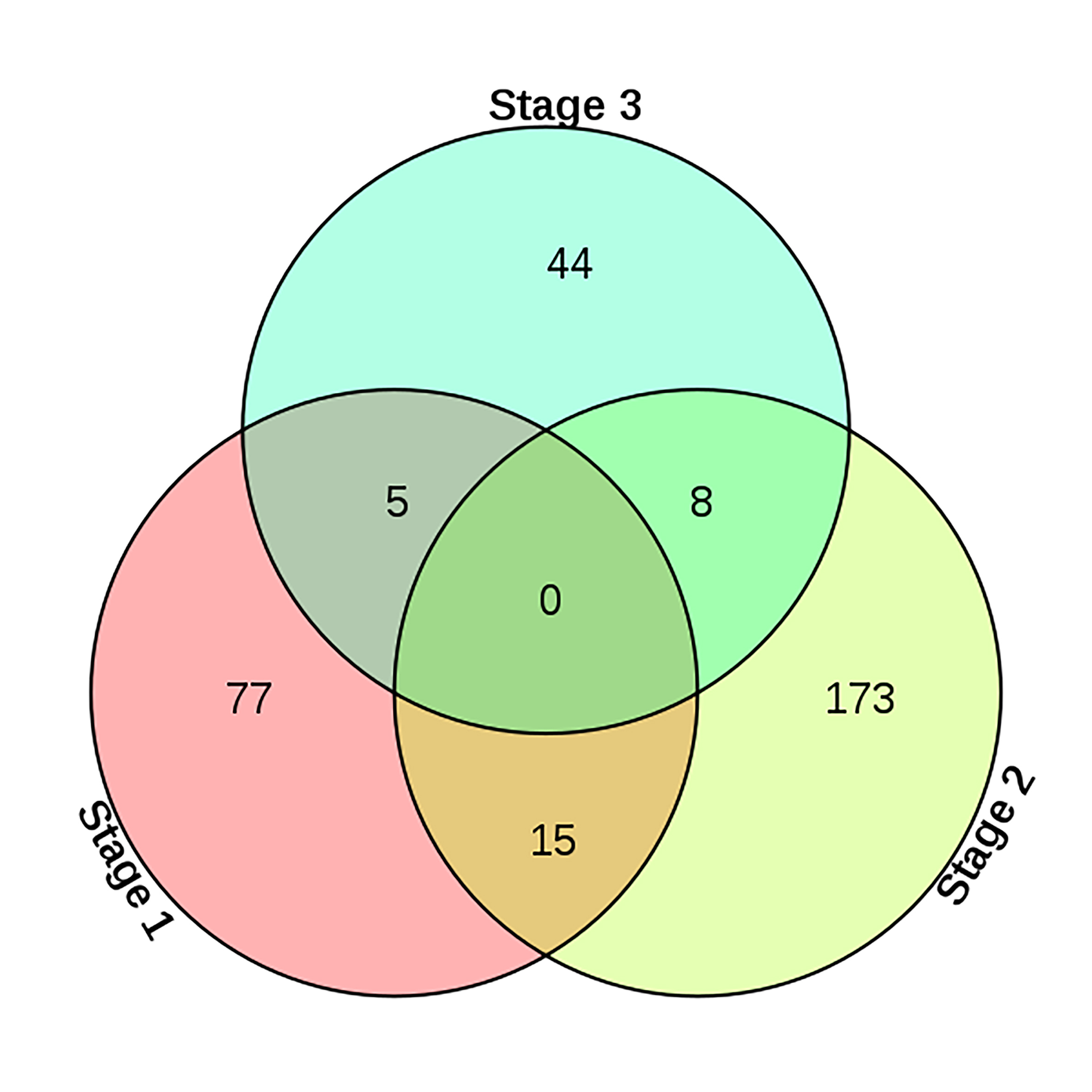

Supplement: Supplementary file 2 — Additional file 2: Fig supplement 2. Venn chart of SDECs detected in three transitional stages. [file 12864_2020_6649_MOESM2_ESM.png]
